# Green ILC: A Novel Energy-Efficient Iterative Learning Control Approach

**DOI:** 10.3390/s24237787

**Published:** 2024-12-05

**Authors:** Yu Dou, Emmanuel Prempain

**Affiliations:** School of Engineering, University of Leicester, Leicester LE1 7RH, UK; yd116@leicester.ac.uk

**Keywords:** iterative learning control, gradient descent optimization, energy-efficient control systems, industrial energy optimization, hybrid control methodologies

## Abstract

In this paper, we introduce Green Iterative Learning Control (Green ILC), an innovative hybrid control method that addresses the critical need for energy-efficient control in dynamic, repetitive-task environments. By integrating the iterative refinement capabilities of traditional Iterative Learning Control (ILC) with the optimization strengths of gradient descent, Green ILC achieves a balanced trade-off between tracking accuracy and energy consumption. This novel approach introduces a cost function that minimizes both tracking errors and control effort, enabling the system to adaptively optimize performance over iterations. Theoretical analysis and simulation results demonstrate that Green ILC not only achieves faster convergence but also provides significant energy savings compared with traditional ILC methods. Notably, Green ILC reduces energy consumption by prioritizing efficiency, making it particularly suitable for energy-intensive applications such as robotics, manufacturing, and process control. While a slight decrease in tracking accuracy is observed, this trade-off is acceptable for scenarios where energy efficiency is paramount. This work establishes Green ILC as a promising solution for modern industrial systems requiring robust and sustainable control strategies.

## 1. Introduction

In the field of modern control engineering, with the rapid development of industrial automation, robotics, and smart manufacturing, control systems are facing increasingly complex challenges [1]. These systems not only need to achieve high precision and performance in complicated operational environments, but they must also possess strong adaptability and robustness to handle constantly changing external conditions and various uncertainties. They must operate seamlessly in environments that are both complex and subject to frequent and unpredictable changes. High precision and performance are crucial, especially in applications such as aerospace, automotive manufacturing, and medical robotics, where even the slightest deviation can have significant consequences. However, achieving these goals is only part of the challenge. Modern control systems must also possess strong adaptability and robustness, which are essential to maintaining optimal performance in the face of fluctuating environmental conditions, unknown disturbances, and system uncertainties.

This adaptability is particularly important in dynamic environments, where variables such as load, temperature, and external forces may change unpredictably. Robustness ensures that control systems can continue to operate effectively in the presence of noise, sensor errors, or inaccuracies in modeling. Although traditional control methods, such as PID controllers, are widely used due to their simplicity and effectiveness in linear, time-invariant systems, they often struggle to meet the demands of more advanced applications that require real-time adjustments and learning capabilities [2]. Another widely used control method is Linear Quadratic Regulator (LQR), which provides optimal control strategies by balancing system performance and control energy. LQR achieves this by minimizing a quadratic cost function that penalizes state errors and control effort [3,4,5]. While LQR is highly effective for linear systems with well-defined models, it has limitations when applied to more complex, nonlinear, or time-varying systems. Its assumption of linearity limits its use in many real-world situations where system dynamics can change unpredictably. These conventional methods rely on fixed parameters, which can limit their effectiveness in situations where adaptability and continuous improvement are needed. In highly complex systems, traditional controllers may struggle to handle nonlinearity, time-varying dynamics, or external disturbances, resulting in performance degradation and operational inefficiency.

More advanced control strategies with learning abilities have become essential to overcoming these limitations [6,7,8]. Among these approaches, Iterative Learning Control (ILC) has gained considerable attention due to its ability to improve performance over time by using data from previous cycles. This makes ILC well suited for systems that perform repetitive tasks, such as robotic arms, automated manufacturing processes, or any system that repeats operations in each cycle [9,10,11,12]. By adjusting control actions based on past results, ILC gradually reduces tracking errors, improving the system’s accuracy and efficiency. However, despite its advantages, the performance of traditional ILC can decline significantly in dynamic or unpredictable environments, as it typically relies on a fixed system model that may not account for changes or disturbances.

The study by Cao et al. introduced modified P-type indirect ILC designed to handle nonlinear systems with mismatched uncertainties and matched disturbances [13]. The method employs a learning-based disturbance estimator to transform the system into an input-to-state stable form, enabling compensation for dynamic mismatched uncertainties. Additionally, modified P-type ILC is implemented to enhance tracking performance for non-repetitive trajectories. Experimental results demonstrate the effectiveness of this approach, with tracking errors being significantly reduced in complex systems such as continuous stirred tank reactors and flight simulators.

In addition to ILC, gradient descent is another well-known optimization method widely used to fine-tune system parameters by minimizing a cost function [14]. This cost function typically includes essential factors such as output error, control energy, and system stability. Gradient descent is commonly used across various fields due to its general applicability. However, in control systems, gradient descent is typically applied non-iteratively, focusing solely on optimizing current parameters without fully utilizing feedback from previous cycles. This lack of iterative learning limits gradient descent’s effectiveness in dynamic environments where continuous adjustments are necessary. While gradient descent works well for optimizing single objectives in relatively stable settings, it becomes less effective in control systems that require adaptation and iterative refinement.

Although ILC and gradient descent differ in many ways, they share similar strategies in updating control inputs and system parameters iteratively to improve performance. This similarity has led researchers to explore combining the strengths of both ILC and gradient descent to create hybrid control methods that address the limitations of each approach. By merging the iterative learning features of ILC with the optimization properties of gradient descent, these hybrid strategies can improve the adaptability and robustness of control systems, especially in environments that experience frequent changes or disturbances.

Sago and Adachi presented a gradient-based ILC algorithm for linear discrete-time systems which addresses the limitations of existing ILC schemes. It provides convergence conditions based on system uncertainties, ensuring exponential decay of input error and improving applicability in uncertain systems [15]. Owens et al. introduced a robust gradient-based ILC algorithm and provided conditions for monotonic convergence in the presence of model uncertainties. This approach ensures that tracking errors decrease consistently, making it suitable for dynamic environments. Their work addresses the practical challenges of modeling errors, contributing to the development of more reliable and adaptable control systems [16]. Chu et al. proposed a predictive gradient-based ILC algorithm that extends traditional ILC by incorporating predicted future trials in addition to past trial data. This approach significantly improves convergence speed and robustness to model uncertainties, offering better predictive control capabilities [17]. Huo et al. presented a model-free gradient-based ILC algorithm for nonlinear systems, eliminating the need for system model identification. The algorithm achieves monotonic convergence to zero error and matches the performance of model-based ILC in stroke rehabilitation, with better tracking than linear model-free ILC [18]. He and Pu proposed a novel gradient-based ILC algorithm to enhance proportional-type traditional ILC by addressing its parameter sensitivity. Their algorithm mimics the gradient descent process, ensuring reliable convergence and reducing parameter dependence, making it more robust than traditional ILC [19]. Developing these hybrid methods offers a promising approach to improving system performance in complex, real-world applications.

Different from the common control objective, which focuses on minimizing the error between actual and desired outputs, this paper also considers the effect of input energy consumption. Drawing inspiration from LQR’s approach to optimizing performance, we aim to extend these principles by integrating the iterative learning capabilities of ILC with the optimization strengths of gradient descent.

This paper introduces Green ILC, a novel hybrid control strategy that combines the characteristics of ILC with the optimization strengths of gradient descent. Green ILC is designed to balance tracking accuracy and energy use through a cost function that minimizes both tracking error and control effort. By using gradient-based updates, Green ILC iteratively improves control inputs over time while optimizing energy consumption. This makes Green ILC particularly useful in applications where energy efficiency is critical, such as robotics, manufacturing, and industrial process control. By combining iterative learning and optimization, Green ILC offers a more adaptable and efficient solution for control systems in dynamic environments, significantly reducing energy consumption while maintaining satisfactory tracking performance.

The rest of this paper is organized as follows: Section 2 explains the theoretical background and mathematical development of Green ILC. Section 3 presents the simulation results, demonstrating its performance and comparing it with traditional ILC. Section 4 discusses Green ILC’s performance in detail, and Section 5 concludes the paper by summarizing the key contributions of this research.

## 2. Method

This section derives the Green ILC updating rule and provides its convergence proof.

### 2.1. Green Iterative Learning Control

Consider the discrete-time state-space model described by the following equations:

(1) $$\begin{array}{cc} x_{j}\left(k+1\right) & =Ax_{j}\left(k\right)+Bu_{j}\left(k\right)+w\left(k\right),\\ y_{j}\left(k\right) & =Cx_{j}\left(k\right), \end{array}$$

where j∈{0,1,2,…} is the iteration index and k∈{0,1,…,N−1} represents the time step within each iteration. Here, $x_{j}\left(k\right)\in R^{n}$ is the state vector, $u_{j}\left(k\right)\in R^{m}$ is the control input vector, and $y_{j}\left(k\right)\in R^{p}$ is the output vector. The disturbance vector $w\left(k\right)\in R^{n}$ is assumed to be iteration-invariant. The system matrices are given by $A\in R^{n\times n}$, $B\in R^{n\times m}$, and $C\in R^{p\times n}$.

The control objective is to find a sequence of control inputs $\left\{u_{j}\left(k\right)\right\}$ that minimizes the following cost function over iterations:

(2) $$J\left(u_{j}\right)=\sum_{k=0}^{N-1}\left(e_{j}{\left(k+1\right)}^{⊤}Qe_{j}\left(k+1\right)+u_{j}{\left(k\right)}^{⊤}Ru_{j}\left(k\right)\right),$$

where $e_{j}\left(k+1\right)=y_{r}\left(k+1\right)-y_{j}\left(k+1\right)$ denotes the tracking error at time step k+1, $y_{r}\left(k+1\right)$ is the desired reference output, and $Q\in R^{p\times p}$ and $R\in R^{m\times m}$ are positive definite weighting matrices (Q > 0 and R > 0).

We substitute the dynamics of the output $y_{j}\left(k+1\right)$ from the system equations

(3) $$\begin{array}{cc} y_{j}\left(k+1\right) & =C\left(Ax_{j}\left(k\right)+Bu_{j}\left(k\right)+w\left(k\right)\right)\\ \; & =CAx_{j}\left(k\right)+CBu_{j}\left(k\right)+Cw\left(k\right), \end{array}$$

which leads to the expression for the error term:

(4) $$\begin{array}{cc} e_{j}\left(k+1\right) & =y_{r}\left(k+1\right)-CAx_{j}\left(k\right)-CBu_{j}\left(k\right)-Cw\left(k\right). \end{array}$$

To minimize the cost function $J(u_{j})$ by using gradient-based methods, we compute the gradient of *J* for $u_{j}\left(k\right)$. We define

(5) $$J_{1}=e_{j}{\left(k+1\right)}^{⊤}Qe_{j}\left(k+1\right),\;J_{2}=u_{j}{\left(k\right)}^{⊤}Ru_{j}\left(k\right).$$

The gradient of $J_{1}$ with respect to $u_{j}\left(k\right)$ is given by the chain rule (see Theorem A1 in Appendix A):

(6) $$\begin{array}{cc} \nabla_{u_{j}\left(k\right)}J_{1} & ={\left(\frac{\partial e_{j}\left(k+1\right)}{\partial u_{j}\left(k\right)}\right)}^{⊤}2Qe_{j}\left(k+1\right)\\ \; & ={\left(-CB\right)}^{⊤}2Qe_{j}\left(k+1\right)\\ \; & =-2B^{⊤}C^{⊤}Qe_{j}\left(k+1\right). \end{array}$$

The gradient of $J_{2}$ is

(7) $$\nabla_{u_{j}\left(k\right)}J_{2}=2Ru_{j}\left(k\right).$$

Thus, the gradient of the cost function *J* is

(8) $$\begin{array}{c} \nabla_{u_{j}\left(k\right)}J=-2B^{⊤}C^{⊤}Qe_{j}\left(k+1\right)+2Ru_{j}\left(k\right). \end{array}$$

By using the gradient descent method (see Theorem A2 in Appendix A) to update the control input, the update rule becomes

(9) $$\begin{array}{cc} u_{j+1}\left(k\right) & =u_{j}\left(k\right)-\alpha \nabla_{u_{j}\left(k\right)}J\\ \; & =u_{j}\left(k\right)-\alpha \left(-2B^{⊤}C^{⊤}Qe_{j}\left(k+1\right)+2Ru_{j}\left(k\right)\right)\\ \; & =\left(I-2\alpha R\right)u_{j}\left(k\right)+2\alpha B^{⊤}C^{⊤}Qe_{j}\left(k+1\right), \end{array}$$

where α is the step size and I is the identity matrix of appropriate dimensions. This update rule shows that each iteration refines the control input to reduce the tracking error, reflecting the iterative learning nature of ILC.

### 2.2. Convergence Proof

To establish the convergence of the Green ILC algorithm, we analyze whether the control inputs $u_{j}\left(k\right)$ converge to an optimal solution $u^{*}\left(k\right)$. For this proof, the following assumptions are made:

- The disturbance w(k) is iteration-invariant.
- The initial state $x_{j}\left(0\right)=x_{0}$ is consistent across all iterations.
- The system is controllable and observable.
- The matrix A is stable or can be stabilized.

To examine how the cost function $J(u_{j})$ evolves, we use a second-order Taylor series expansion (see Theorem A3 in Appendix A) around $u_{j}\left(k\right)$:

(10) $$J\left(u_{j+1}\right)\approx J\left(u_{j}\right)+\nabla_{u_{j}}J^{⊤}\left(u_{j+1}-u_{j}\right)+\frac{1}{2}{\left(u_{j+1}-u_{j}\right)}^{⊤}\nabla_{u_{j}}^{2}J\left(u_{j+1}-u_{j}\right).$$

Substituting the gradient descent update rule $u_{j+1}-u_{j}=-\alpha \nabla_{u_{j}}J$ into this expansion yields

(11) $$J(u_{j+1})\approx J\left(u_{j}\right)-\alpha \nabla_{u_{j}}J^{⊤}\nabla_{u_{j}}J+\frac{\alpha^{2}}{2}\nabla_{u_{j}}J^{⊤}\nabla_{u_{j}}^{2}J\nabla_{u_{j}}J.$$

The change in the cost function, ΔJ, is

(12) $$\Delta J=J\left(u_{j+1}\right)-J\left(u_{j}\right)\approx -\alpha {∥\nabla_{u_{j}}J∥}^{2}+\frac{\alpha^{2}}{2}\nabla_{u_{j}}J^{⊤}\nabla_{u_{j}}^{2}J\nabla_{u_{j}}J.$$

By assuming the maximum eigenvalue *L* of the Hessian $\nabla_{u_{j}}^{2}J$, the second-order term can be bounded by the Rayleigh Quotient inequality (see Theorem A4 in Appendix A):

(13) $$\nabla_{u_{j}}J^{⊤}\nabla_{u_{j}}^{2}J\nabla_{u_{j}}J\leq L{∥\nabla_{u_{j}}J∥}^{2}.$$

We substitute this into the expression for ΔJ:

(14) $$\Delta J\leq -\left(\alpha -\frac{\alpha^{2}L}{2}\right){∥\nabla_{u_{j}}J∥}^{2}.$$

For a guaranteed decrease in the cost function, the following condition must hold:

(15) $$0<\alpha <\frac{2}{L}.$$

This condition ensures that the update rule results in a monotonic decrease in the cost function $J(u_{j})$, leading to the convergence of the control inputs $u_{j}\left(k\right)$ toward the optimal solution $u^{*}\left(k\right)$.

## 3. Results

To demonstrate the effectiveness of the proposed Green ILC method, we test it on three numerical cases.

### 3.1. Case 1: Sinusoidal Trajectory

First, we consider a discrete-time system characterized by the following state-space matrices:

(16) $$A=\left[\begin{array}{cc} 0.1 & 0.7\\ 0 & 0.5 \end{array}\right],\;B=\left[\begin{array}{c} 1\\ 0 \end{array}\right],\;C=\left[\begin{array}{cc} 1 & 0 \end{array}\right].$$

The control objective is to track a reference trajectory while minimizing the cost function defined in Equation (2). The reference trajectory is defined as

(17) $$y_{r}\left(k\right)=\sin\left(\frac{2\pi k}{N}\right),$$

for N=50 time steps. The external disturbance is defined as

(18) $$w\left(k\right)=0.05\sin\left(\frac{k}{10}\right).$$

The Green ILC method is applied over 30 iterations, with a gradient descent step size of α=0.025. The control input is updated iteratively according to the rule in Equation (9). The weighting scalars are set to Q=20 and R=1. For comparison, traditional ILC uses learning gains of 0.1, 0.5, and 1.

**Figure 1:**
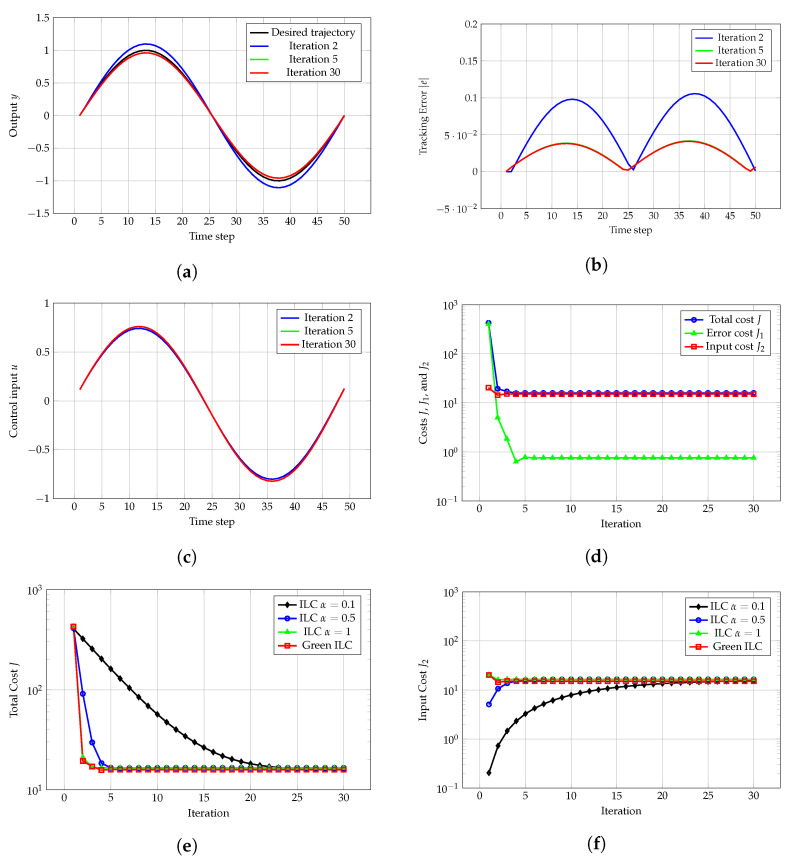
(**a**) Desired trajectory $y_{r}$ and actual trajectories *y* at the 2nd, 5th, and 30th iterations. (**b**) Tracking error |e| at the 2nd, 5th, and 30th iterations. (**c**) Control input *u* at the 2nd, 5th, and 30th iterations. (**d**) Total cost *J*, error cost $J_{1}$, and input cost $J_{2}$ for Green ILC over iterations. (**e**) Comparison of total cost *J* for traditional ILC and Green ILC over iterations. (**f**) Comparison of input cost $J_{2}$ for traditional ILC and Green ILC over iterations.

Figure 1a compares the desired trajectory $y_{r}$ and the actual trajectories *y* at the 2nd, 5th, and 30th iterations. The results show near-perfect tracking with minimal errors at the 30th iteration, demonstrating Green ILC’s ability to optimize both tracking accuracy and control energy. Although Green ILC may result in slightly larger tracking errors than traditional ILC, the difference is minor when high precision is not critical. This trade-off highlights Green ILC’s capacity to balance accuracy with energy efficiency, making it ideal for applications where energy savings are prioritized over extreme precision.

Figure 1b shows the tracking error |e| at the 2nd, 5th, and 30th iterations, with a clear reduction in error across iterations. The tracking error |e| remains almost unchanged after the fifth iteration, indicating that the system has reached equilibrium.

Figure 1c compares the control input *u* at the 2nd, 5th, and 30th iterations, revealing that the inputs are very similar, indicating the system output’s high sensitivity to input variations. This means that tiny variations in the input might lead to large differences in the output.

Figure 1d displays the total cost *J*, output error cost $J_{1}$, and control input cost $J_{2}$ over iterations. After approximately five iterations, all three costs reach equilibrium, indicating that the system has effectively balanced tracking accuracy with control input energy, thereby validating the convergence of the Green ILC method.

Figure 1e shows the total cost *J* for both Green ILC and traditional ILC over iterations. Green ILC stabilizes after 4 iterations, whereas traditional ILC requires 25, 5, or 4 iterations for different learning gains. At equilibrium, the total cost *J* for Green ILC (15.88 units) is lower than for traditional ILC (16.01, 16.52, or 16.52 units).

Figure 1f illustrates the control input energy consumption over iterations for both traditional ILC and Green ILC. Since Green ILC explicitly considers input energy in its optimization, it results in lower energy consumption in the steady state. After reaching equilibrium, Green ILC requires only 15.13 units of energy, compared with 15.57, 16.52, and 16.52 units for traditional ILC. This reduction in control effort underscores Green ILC’s ability to maintain desired performance while minimizing energy consumption.

### 3.2. Case 2: Random Trajectory

Second, we consider another discrete-time system with the following state-space matrices:

(19) $$A=\left[\begin{array}{cc} 0.9 & 0.3\\ 0 & 0.7 \end{array}\right],\;B=\left[\begin{array}{c} 1\\ 0 \end{array}\right],\;C=\left[\begin{array}{cc} 1 & 0 \end{array}\right].$$

To increase the difficulty of the tracking task, a random signal is used as the reference trajectory, serving as a robust test for the proposed method. Additionally, random noise, constant at each iteration, is added to evaluate the learning capability of Green ILC.

The Green ILC method is applied over 60 iterations, with a gradient descent step size of α=0.015. The control input is updated iteratively according to the rule in Equation (9). The weighting scalars are set to Q=5 and R=1. For comparison, traditional ILC uses learning gains of 0.1, 0.2, and 0.25.

**Figure 2:**
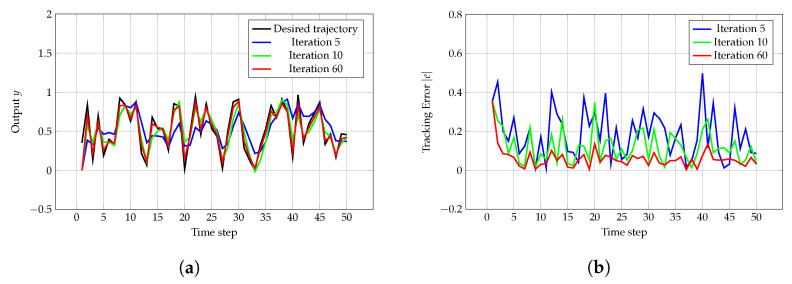

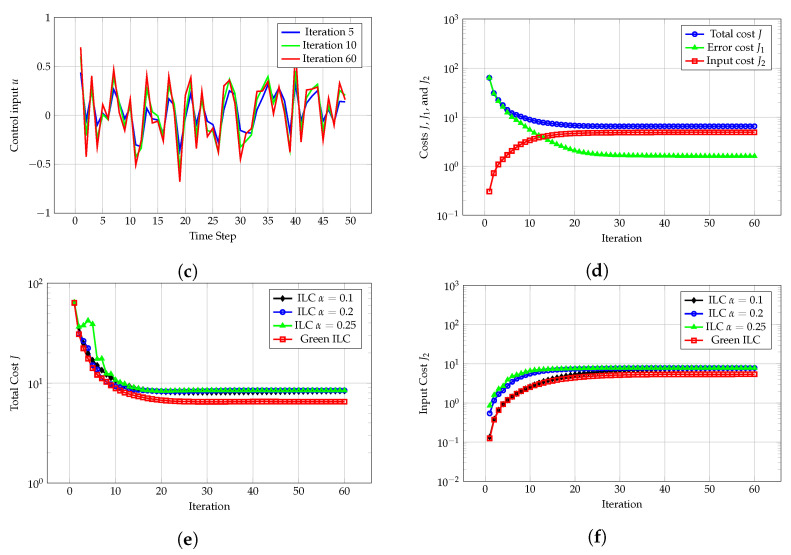
(**a**) Desired trajectory $y_{r}$ and actual trajectories *y* at the 5th, 10th, and 60th iterations. (**b**) Tracking error |e| at the 5th, 10th, and 60th iterations. (**c**) Control input *u* at the 5th, 10th, and 60th iterations. (**d**) Total cost *J*, error cost $J_{1}$, and input cost $J_{2}$ for Green ILC over iterations. (**e**) Comparison of total cost *J* for traditional ILC and Green ILC over iterations. (**f**) Comparison of input cost $J_{2}$ for traditional ILC and Green ILC over iterations.

Figure 2a–d illustrate a similar phenomenon as observed in Case 1. Figure 2e demonstrates that Green ILC stabilizes after 30 iterations, whereas traditional ILC achieves stabilization in only 20, 20, or 20 iterations. However, at equilibrium, the total cost *J* for Green ILC (6.53 units) is lower than that for traditional ILC (8.35, 8.46, or 8.46 units), underscoring the superior optimization capabilities of Green ILC. Figure 2f shows that after reaching equilibrium, Green ILC requires only 5.4 units of energy, in contrast to the 7.70, 7.83, or 7.84 units consumed by traditional ILC. The more pronounced energy reduction in Case 2 compared with Case 1 is attributed to the decrease in the error weight *Q*.

### 3.3. Case 3: Complex Trajectory

Thirdly, we consider a discrete-time system characterized by the following 3×3 state-space matrices:

(20) $$A=\left[\begin{array}{ccc} 0.1 & 0.7 & 0\\ 0 & 0.5 & 0\\ 0 & 0 & 0.5 \end{array}\right],\;B=\left[\begin{array}{c} 1\\ 0\\ 1 \end{array}\right],\;C=\left[\begin{array}{ccc} 1 & 0 & 1 \end{array}\right].$$

The reference trajectory is defined as

(21) $$y_{r}\left(k\right)=\sin\left(\frac{2\pi k}{N}\right)+\sin\left(\frac{10\pi k}{N}\right),$$

for N=50 time steps. The external disturbance is defined as in Equation (18). The Green ILC method is applied over 60 iterations, with a gradient descent step size of α=0.02. The control input is updated iteratively according to the rule in Equation (9). The weighting scalars are set to Q=5 and R=1. For comparison, traditional ILC uses learning gains of 0.1, 0.5, and 0.6.

**Figure 3:**
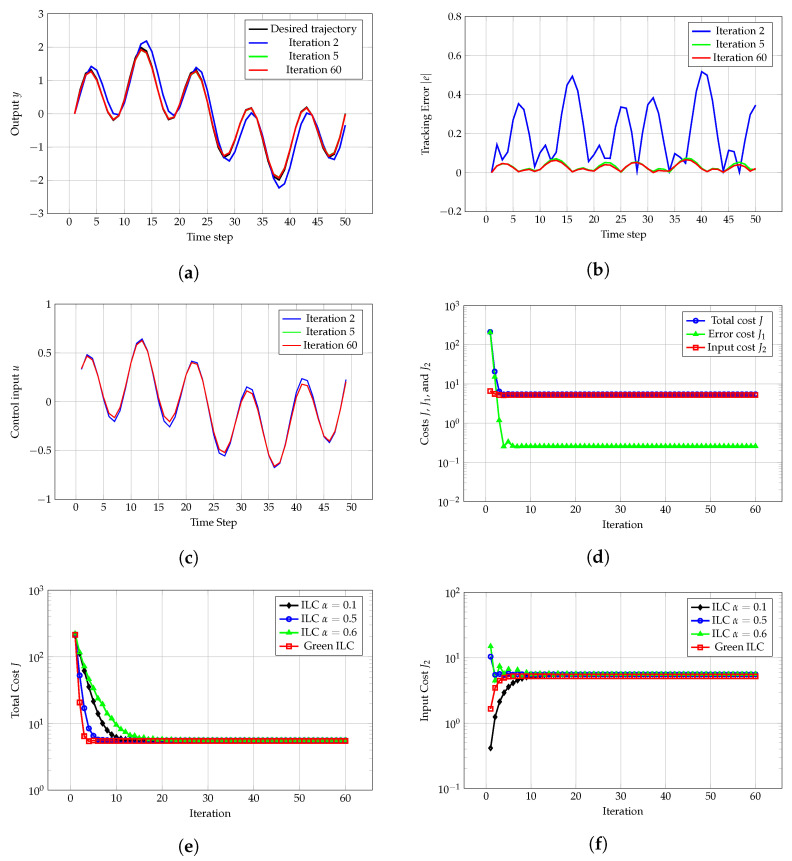
(**a**) Desired trajectory $y_{r}$ and actual trajectories *y* at the 2nd, 5th, and 60th iterations. (**b**) Tracking error |e| at the 2nd, 5th, and 60th iterations. (**c**) Control input *u* at the 2nd, 5th, and 60th iterations. (**d**) Total cost *J*, error cost $J_{1}$, and input cost $J_{2}$ for Green ILC over iterations. (**e**) Comparison of total cost *J* for traditional ILC and Green ILC over iterations. (**f**) Comparison of input cost $J_{2}$ for traditional ILC and Green ILC over iterations.

Figure 3a–d show similar performance to the previous cases. Figure 3e shows that Green ILC stabilizes after 30 iterations, whereas traditional ILC requires the same as in the previous cases. At equilibrium, the total cost *J* for Green ILC (5.49 units) is lower than that for traditional ILC (5.62, 5.62, or 5.62 units). Figure 3f illustrates that after reaching equilibrium, Green ILC requires only 5.23 units of energy, compared with 5.62, 5.62, or 5.62 units used by traditional ILC.

## 4. Discussion

Green ILC combines the strengths of gradient descent optimization and the ILC strategy, leading to significant reductions in energy consumption while maintaining good system performance. However, compared with traditional ILC, Green ILC may show a slight decrease in tracking accuracy. This method optimizes control inputs and tracking errors, prioritizing energy efficiency over precision. As a result, it may not be the best choice for applications where high precision is crucial, but it is highly effective in cases where managing energy use is the main concern.

The main advantage of Green ILC is its ability to significantly reduce control energy consumption, especially in energy-intensive systems. By balancing tracking accuracy with control inputs, Green ILC helps avoid excessive energy usage, which can lead to actuator wear or failure. In many repetitive-task applications, such as robotics, automated manufacturing, and process control, Green ILC provides acceptable tracking accuracy while saving energy by reducing control effort. Additionally, Green ILC can handle certain unknown disturbances that traditional LQR methods cannot manage, increasing its robustness and expanding its range of practical applications.

In Green ILC, the weighting matrices Q and R are pivotal in balancing tracking errors and control energy. By dynamically adjusting these matrices based on system states and performance requirements, Green ILC achieves adaptive optimization of control strategies, thereby enhancing system robustness and reliability. For instance, in robotics, Green ILC can modify the weighting matrices to meet specific task demands, such as precise assembly or high-speed material handling. In manufacturing, it optimizes energy consumption and efficiency across production lines, accommodating various product specifications. In Unmanned Aerial Vehicles (UAVs), Green ILC’s dynamic adjustment of Q and R significantly improves system reliability and broadens its application scope. During missions in complex terrains or under variable weather conditions, increasing the weight of Q emphasizes trajectory tracking accuracy, ensuring flight safety. Conversely, for long-distance missions, elevating the weight of R reduces energy consumption, thereby extending flight duration. This flexibility allows Green ILC to adapt to diverse scenarios, including logistics, environmental monitoring, and disaster relief, thereby enhancing UAV system reliability and versatility. In aerospace applications, Green ILC demonstrates substantial potential. During satellite deployment, increasing the weight of Q ensures precise positioning. For extended space missions, adjusting R to lower energy consumption can prolong operational lifespan. This adaptability makes Green ILC suitable for various aerospace tasks, such as satellite orbit control, spacecraft docking, and planetary exploration, thereby improving system reliability and expanding its range of applications.

Furthermore, Green ILC addresses sensitivity to initial conditions through its adaptive learning mechanism. By integrating the iterative refinement capabilities of traditional ILC with the optimization strengths of gradient descent, Green ILC dynamically adjusts control inputs based on feedback from previous iterations. This adaptive process allows the system to progressively correct deviations caused by suboptimal initial conditions, leading to improved convergence and performance over time. As a result, Green ILC reduces the impact of initial state variations, ensuring robust and consistent control outcomes across different starting points.

However, in high-dimensional nonlinear systems, ILC methods, including Green ILC, can encounter challenges related to computational efficiency and convergence speed. For instance, the study [20] examines the difficulties in achieving robust convergence in the presence of non-repetitive uncertainties, underscoring the limitations of ILC approaches in complex nonlinear environments.

To assess actuator reliability and safety, it is essential to evaluate actuator wear by assessing input energy variance and thermal stability. High variance in input energy indicates fluctuating loads, leading to increased wear. Monitoring this variance provides insights into the actuator’s degree of wear. Green ILC optimizes control strategies to reduce energy consumption, thereby decreasing load fluctuations and operating temperatures. This reduction not only minimizes wear but also enhances thermal stability, indirectly extending the actuator’s service life and improving reliability and safety.

Both papers [18,19] provide experimental results confirming the advantages of gradient-based ILC methods, supporting the performance improvements introduced by Green ILC. The paper [18] validates a model-free gradient ILC in stroke rehabilitation tasks, achieving monotonic convergence with tracking errors up to 1000 times smaller than traditional linear model-free ILC methods. The paper [19] demonstrates a reduction in the standard deviation of normalized steady-state errors from 0.224 in traditional proportional ILC to 0.009 in gradient-based ILC, showcasing enhanced robustness and adaptability for nonlinear systems. These findings, together with the energy optimization and tracking improvements of Green ILC, underscore the ability of gradient-based methods to outperform traditional ILC by enhancing convergence, reducing errors, and addressing energy efficiency in complex control problems.

The Green ILC method is based on the assumption of a known linear model with fixed matrices A, B, and C. While this framework offers mathematical simplicity and ensures convergence, it restricts the method’s applicability to systems with nonlinear or uncertain dynamics. In practical applications, linear models are often used as approximations of system behavior within specific operating ranges. To overcome these limitations, future research could focus on developing adaptive or model-free extensions, which would allow the method to handle time-varying or nonlinear systems effectively. Furthermore, integrating Green ILC with robust control strategies or predictive techniques could significantly enhance its applicability and adaptability in dynamic and complex environments.

## 5. Conclusions

In this paper, Green ILC is introduced as an innovative hybrid control strategy that effectively optimizes energy consumption while maintaining satisfactory tracking accuracy. By combining the principles of gradient descent optimization with iterative learning, the algorithm delivers significant improvements in convergence speed and energy efficiency compared with traditional ILC methods. Simulation results highlight reductions in energy usage and enhanced robustness in dynamic environments.

The practical applications of Green ILC are extensive, spanning robotic manipulators, UAVs, and aerospace systems, where energy efficiency and system reliability are of paramount importance. To maximize its potential in power system design, adaptive weighting strategies should be employed to minimize energy fluctuations and reduce actuator wear. Integrating Green ILC with renewable energy systems could improve stability under variable power conditions, making it a valuable solution for sustainable energy management.

Future research should focus on extending the applicability of Green ILC to nonlinear and time-varying systems, incorporating predictive models to enhance adaptability, and validating its performance in real-world scenarios. These advancements would further establish Green ILC as a practical approach to addressing energy-efficient control challenges.

## Data Availability

The data used to support this research article are available from the author upon request.

## Appendix A

**Theorem** **A1** (Chain Rule [21])**.** *Let $f:R^{m}\to R^{n}$ and $g:R^{n}\to R$ be differentiable functions. Let us define the composite function $h:R^{m}\to R$ by h(x)=g(f(x)), where $x\in R^{m}$ and y=f(x). Then, the gradient of h with respect to x is given by*

(A1) ∇h(x)=∇g(y)·∇f(x),

*where ∇g(y) is the gradient of g with respect to y and ∇f(x) is the Jacobian matrix of f with respect to x.*

**Theorem** **A2** (Gradient Descent Method [22])**.** *Let $f:R^{n}\to R$ be a differentiable function. The gradient descent algorithm updates the parameter $x\in R^{n}$ iteratively to find a local minimum of f. The update rule is given by*

(A2) $$x_{k+1}=x_{k}-\alpha \nabla f\left(x_{k}\right),$$

*where α>0 is the step size and $\nabla f(x_{k})$ is the gradient of f with respect to x at iteration k.*

**Theorem** **A3** (Taylor Series Expansion [23])**.** *Let $f:R^{n}\to R$ be a twice continuously differentiable function at $x_{0}$. The second-order Taylor expansion of f around $x_{0}$ is given by*

(A3) $$f\left(x\right)\approx f\left(x_{0}\right)+\nabla f{\left(x_{0}\right)}^{⊤}\left(x-x_{0}\right)+\frac{1}{2}{\left(x-x_{0}\right)}^{⊤}\nabla^{2}f\left(x_{0}\right)\left(x-x_{0}\right),$$

*where $\nabla f(x_{0})$ is the gradient of f at $x_{0}$ and $\nabla^{2}f\left(x_{0}\right)$ is the Hessian matrix of f at $x_{0}$.*

**Theorem** **A4** (Rayleigh Quotient Inequality [24])**.** *Let $H\in R^{n\times n}$ be a symmetric positive definite matrix, and let $z\in R^{n}$ be a non-zero vector. The quadratic form $z^{⊤}Hz$ satisfies the inequality*

(A4) $$\lambda_{\min}\left(H\right){∥z∥}^{2}\leq z^{⊤}Hz\leq \lambda_{\max}\left(H\right){∥z∥}^{2},$$

*where $\lambda_{\min}\left(H\right)$ and $\lambda_{\max}\left(H\right)$ denote the smallest and largest eigenvalues of H, respectively.*
